# Metformin use and lung cancer survival: a population-based study in Norway

**DOI:** 10.1038/s41416-020-01186-9

**Published:** 2020-12-02

**Authors:** Suzan Brancher, Nathalie C. Støer, Elisabete Weiderpass, Ronald A. M. Damhuis, Tom B. Johannesen, Edoardo Botteri, Trond-Eirik Strand

**Affiliations:** 1 Department of Epidemiology, School of Public Health, University of São Paulo, São Paulo, Brazil; 2 Norwegian National Advisory Unit on Women’s Health, Women’s Clinic, Oslo University Hospital, Oslo, Norway; 3 Cancer Registry of Norway, Oslo University Hospital, Oslo, Norway; 4 International Agency for Research on Cancer (IARC), World Health Organization, Lyon, France; 5 Department of Research, Comprehensive Cancer Organization, Utrecht, The Netherlands

**Keywords:** Non-small-cell lung cancer, Small-cell lung cancer, Non-small-cell lung cancer

## Abstract

**Background:**

We assessed associations between metformin use and survival in a nationwide Norwegian cohort of lung cancer (LC) patients.

**Methods:**

The study linked 22,324 LC patients from the Cancer Registry of Norway diagnosed 2005–2014 with the Norwegian Prescription Database. We estimated associations of pre- and post-diagnostic metformin use with overall survival (OS) and LC-specific survival (LCSS) using multivariable time-fixed and time-dependent Cox regression.

**Results:**

Pre-diagnostic metformin use was not associated with improved survival in all patients. Nevertheless, pre-diagnostic metformin use was associated with better LCSS in squamous cell carcinoma (SCC) patients (hazard ratio (HR) = 0.79; 95% confidence interval (CI) 0.62–0.99) and in patients with regional stage SCC (HR = 0.67; 95%CI 0.47–0.95). Post-diagnostic metformin use was associated with improved LCSS in all patients (HR = 0.83; 95%CI 0.73–0.95), in patients with SCC (HR = 0.75; 95%CI 0.57–0.98), regional stage LC (HR = 0.74; 95%CI 0.59–0.94), and regional stage SCC (HR = 0.57; 95%CI 0.38–0.86). OS showed similar results. Analyses of cumulative use showed a dose-response relationship in all patients, patients with adenocarcinoma and SCC, and with regional and metastatic LC.

**Conclusions:**

Metformin use was associated with improved survival, especially LCSS in patients with regional stage SCC. Further prospective studies are required to clarify the role of metformin in LC treatment.

## Background

Identifying new targets for approved drugs traditionally used for non-cancer indications is known as drug repositioning strategy.^1,2^ Metformin, the first-line therapy for type 2 diabetes mellitus (T2D), is one of the most studied repositioning drug due to its safety, inexpensiveness, and well-tolerated profile associated with anti-cancer activity postulated in preclinical studies.^3–5^

Diabetes mellitus (DM) is a chronic condition commonly seen in lung cancer (LC) patients,^6^ and use of metformin as an adjuvant strategy for different LC treatment has been under investigation for years. Several observational studies reported beneficial effects of metformin use on survival in non-small cell lung cancer (NSCLC)^1,6–16^ and small-cell lung cancer (SCLC)^17,18^ in diabetic patients. However, the real benefit of metformin use for LC survival remains unclear due to high heterogeneity between studies, and the possibility of biases that might have led to an overestimated effect of metformin on survival.^19^ Clinical trials were conducted in non-diabetic patients with contradictory findings for the use of metformin in addition to chemotherapy, and combined with targeted therapy for advanced NSCLC patients.^20–24^

Our study intended to assess whether metformin use is associated with overall survival (OS) and lung cancer-specific survival (LCSS) with a population-based design of 22,324 LC patients, properly analysed with a time-dependent approach to reduce the risk of immortal time bias.

## Methods

### Data sources

The Norwegian 11-digit unique personal identification number allowed univocal linkage of population-based health registries. Cancer information was obtained from the Cancer Registry of Norway (CRN). CRN has recorded cancer incidence on a nationwide basis since 1953 with high completeness. CRN is linked to National Population Registry and Cause of Death Registry to obtain vital status and cause of death and receives all reports from pathology departments at Norwegian hospitals. The Norwegian Prescription Database (NorPD) contains detailed individual level information about all prescription drugs dispensed from pharmacies since 2004. Reporting to CRN and NorPD is mandatory by law.

This study was approved by the Regional Ethics Committee in the South East region of Norway (2011/2470/REK Sør-Øst C).

### Study population

We identified 27,354 patients with primary LC diagnosis (Classification of Disease for Oncology, third edition (ICD-O-3) codes C33-C34), diagnosed from 01.01.2005 to 31.12.2014. Histologic subtypes, based on ICD-O-3 morphology codes, were defined as adenocarcinoma (8140/3, 8250/3, 8253/3, 8254/3, 8255/3, 8260/3, 8310/3, 8323/3, 8333/3, 8480/3, 8481/3, 8490/3, 8551/3, 8560/3, 8570/3 and 8574/3), squamous cell carcinoma (SCC) (8052/3, 8070/2, 8070/3, 8071/3, 8072/3, 8073/3, 8074/3, and 8076/3), NSCLC- Not Otherwise Specified (NOS) (8010/3, 8020/3, 8022/3, 8031/3, 8032/3, 8046/3, 8246/3, 8249/3, 8490/3, 8562/3, 8972/3 and 8973/3), large cell carcinoma (8012/3) and small cell carcinoma (SCLC) (8041/3, 8042/3, 8043/3, 8044/3, and 8045/3). Other histologies were classified as “others”, and included carcinoid (8240/3, 8244/3 and 8249/3), pleomorphic carcinoma (8022/3), giant cell carcinoma (8031/3), adenoid cystic carcinoma (8200/3), large-cell neuroendocrine carcinoma (8013/3), and neoplasm malignant, including blastoma NOS (8000/3).

We excluded sarcomas, lymphomas, benign tumours (*n* = 424) and tumours with missing histology (*n* = 3118). Additionally, we excluded patients with date of diagnosis at the date of death (*n* = 1470), patients emigrating before diagnosis (*n* = 10) and patients diagnosed before the age of 20 (*n* = 8). This provided a final study sample of 22,324 LC patients.

### Metformin and other variables

Use of metformin (Anatomical Therapeutic Chemical code A10BA02), between January 2004 and December 2014, was separated into pre-diagnostic and post-diagnostic use. Users of other anti-diabetic medications were classified as insulin users (A10A), users of other blood glucose lowering drugs (A10BB, A10BF, A10BG, A10BH and A10BX), and combination users (A10BD). Metformin combined with other blood glucose lowering drugs was defined as combination use. A table with frequencies of pre-diagnostic anti-diabetic medications is available in the supplementary material (Table S1).

Other variables were demographic characteristics (gender and age categorised as <60, 60–69, 70–79, 80+ years), smoking habits (current, previous, and never smoking), histology, treatment (surgery and radiotherapy) and stage, defined by CRN as localised (TNM I), regional (TNM II and III), and metastatic (TNM IV).^25^ Surgery was classified as not with curative-intent (no surgery or procedures or surgery without complete removal of tumour) and with curative-intent (removal of primary tumour). Radiotherapy was classified as no radiation, radiation with curative intention only and any other intent (unknown, prophylactic, palliative, local control or curative intent radiotherapy administered after any other intention).

### Statistical analysis

Events of interest were all-cause death and lung cancer-specific death. Patients were censored at emigration or end of follow-up (30.06.2015) in addition to death from causes other than LC when studying LCSS.

Kaplan–Meier method and log-rank test were used for crude survival curves for pre-diagnostic use. Cox proportional hazard models were used to estimate hazard ratio (HR) and 95% confidence interval (CI) for the association between OS and LCSS for pre-diagnostic and post-diagnostic use of metformin. Pre-diagnostic metformin use was analysed as a time-fixed variable and post-diagnostic metformin use as a time-dependent variable which reduces the risk of immortal time bias.^26^ Test for interaction was applied in all subgroup analyses.

Pre-diagnostic metformin use was defined as at least one prescription in a one-year window before diagnosis. For post-diagnostic use, the exposure was defined as ≥1 prescription within 3 months before cancer diagnosis to date of death/censoring. Person-time was analysed as unexposed until first use of metformin and exposed from that time until death or censoring, or until they potentially switched to a different anti-diabetic medication. When a patient switched, that person was re-categorised as a combination user and the person-time from that time on was categorised as combination use.

Cumulative dose of post-diagnostic metformin was created as a time-dependent variable by first transforming the number of defined daily doses (DDD) in each prescription to grams and cumulating over prescriptions. Based on this, thresholds were defined as the quartiles of the maximum cumulated metformin doses (1–149 grams (g), 150–339 g, 340–749 g and >749 g). The person-time was analysed as unexposed until the first use of metformin, then as exposed in the lowest cumulative dose category until the accumulated dose crossed the next threshold for which the exposure would change to the corresponding cumulative dose category, and similarly for the following thresholds.

To test the trend in cumulative dose, we analysed the categorical cumulative dose of metformin as a continuous variable.

The reference group for pre-diagnostic use and post-diagnostic use was no-use of any anti-diabetic medication while the reference group for cumulative dose was no-use of metformin. All HRs were adjusted for age at diagnosis, gender, smoking, stage, histology, surgery and radiotherapy except for the stratified analyses by stage and/or histology where the stratification variables were not adjusted for. The associations with cumulative dose of metformin were additionally adjusted for use of other anti-diabetics than metformin.

All statistical analyses were performed using R version 3.5.1 (http://cra.r-project.org).

## Results

We followed 22,324 LC patients diagnosed from 2005–2014 until 30.06.2015 (median follow-up was 8.5 months); 16,928 deaths (75.7%) and 14,755 (66%) LC deaths were observed. Respectively, 560 (2.5%) and 408 (1.8%) were pre- and post-diagnostic metformin users.

Pre-diagnostic metformin users were older at diagnosis, more likely to be male, less likely to be current smokers and were more frequently diagnosed with SCC and SCLC than non-users (Table 1). Post-diagnostic metformin users were older, more likely to be diagnosed with SCC and SCLC, less frequently diagnosed with metastatic stage, more likely to have undergone surgery with curative-intent, and less frequently submitted to radiotherapy treatment than non-users. Characteristics of all anti-diabetic medication users are reported in supplementary Table S2.

**Table 1:** Characteristics of lung cancer patients.

Metformin use	Pre-diagnostic			Post-diagnostic^b^		
Characteristics	No use^a^	Metformin	*P* value	No use^a^	Metformin	*P* value
Total (%)	20565 (92.1)	560 (2.5)		20470 (91.7)	408 (1.8)	
Age (y)			<0.001^c^			<0.001^c^
<60	3944 (19.2)	59 (10.5)		3868 (18.9)	43 (10.5)	
60–69	7051 (34.3)	208 (37.1)		6982 (34.1)	163 (40.0)	
70–79	6718 (32.7)	212 (38.0)		6720 (32.8)	148 (36.3)	
80 and older	2852 (13.9)	81 (14.4)		2900 (14.2)	54 (13.2)	
Mean	68.7	70.5	<0.001	68.8	70.0	0.008
Gender			0.004			0.352
Male	11378 (55.3)	345 (61.6)		11341 (55.4)	236 (57.8)	
Female	9187 (44.7)	215 (38.4)		9129 (44.6)	172 (42.2)	
Smoking			0.006			0.481
Current	8186 (56.9)	196 (49.1)		8124 (56.9)	158 (53.4)	
Previous	4839 (33.6)	163 (40.9)		4805 (33.6)	107 (36.2)	
Never	1360 (9.5)	40 (10.0)		1355 (9.5)	31 (10.5)	
Missing	6180	161		6186	112	
Stage			0.711			<0.001
Localised	4218 (21.3)	113 (20.7)		4164 (21.1)	108 (27.3)	
Regional	6173 (31.1)	179 (32.8)		6124 (31.0)	143 (36.1)	
Metastatic	9441 (47.6)	254 (46.5)		9455 (47.9)	144 (36.5)	
Missing	733	14		727	13	
Histology			0.019^d^			0.008^d^
Adenocarcinoma	8287 (40.3)	198 (35.4)		8235 (40.2)	140 (34.3)	
Squamous	4507 (21.9)	141 (25.2)		4478 (21.9)	116 (28.4)	
NSCLC-NOS	2971 (14.5)	69 (12.3)		2968 (14.5)	54 (13.2)	
Large-cell	391 (1.9)	8 (1.4)		391 (1.9)	3 (0.7)	
Small-cell	3431 (16.7)	114 (20.4)		3428 (16.8)	74 (18.1)	
Others	978 (4.8)	30 (5.4)		970 (4.7)	21 (5.2)	
Surgery			0.449			<0.001
No curative-intent	15480 (75.3)	430 (76.8)		15502 (75.8)	273 (66.9)	
Curative-intent	5079 (24.7)	130 (23.2)		4962 (24.3)	135 (33.1)	
Missing	6	0		6	0	
Radiotherapy			0.214			<0.001
No	10121 (49.2)	291 (52.2)		10159 (49.6)	226 (55.4)	
Curative-intent	1899 (9.2)	56 (10.0)		1881 (9.2)	52 (12.8)	
Other intent	8545 (41.6)	212 (37.9)		8420 (41.2)	130 (31.9)	

*NSCLC-NOS* non-small cell lung cancer not otherwise specified.

*P* values do not include missing category.

^a^No use of any anti-diabetic medication.

^b^Numbers calculated at end of follow-up.

^c^*P* value based on categorical age.

^d^From Fisher’s exact test.

**Fig. 1:**
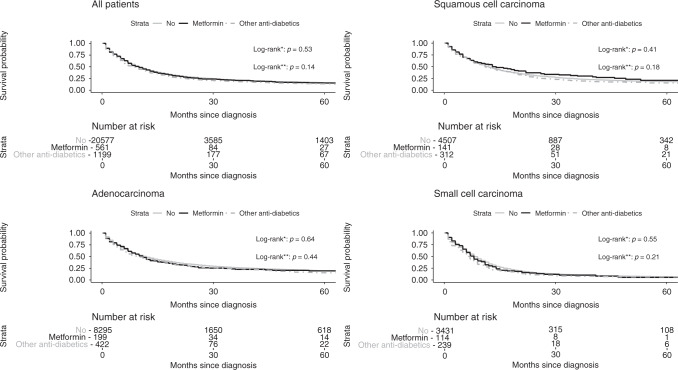
Kaplan–Meier curves for overall survival. Asterisk indicates metformin versus no use. Double asterisk metformin versus any other anti-diabetic medication.

OS was not significantly different between pre-diagnostic metformin users and no-users (*P* = 0.53) nor in metformin users and users of any other anti-diabetic medication (*P* = 0.14), and no differences were observed when stratified by histology (Fig. 1).

Pre-diagnostic metformin use was not associated with improved survival in LC patients as a whole (Table 2). However, when stratified by histological subtypes, an association with metformin use and LCSS for SCC patients was observed (HR = 0.79; 95%CI 0.62–0.99), especially in patients diagnosed with regional stage SCC (HR = 0.67; 95%CI 0.47–0.95). Nonetheless, the interactions with histology (*P* = 0.547) and stage (*P* = 0.442) were not significant.

**Table 2:** Pre-diagnostic metformin use compared to no use of any anti-diabetic medication for all-cause and lung cancer-specific mortality.

Variables	All-cause death	Lung cancer death
	HR (95%CI)	HR (95%CI)
All patients^a^	0.97 (0.88–1.08)	0.95 (0.86–1.06)
Histology^b^		
Adenocarcinoma	1.06 (0.89–1.25)	1.05 (0.87–1.26)
Squamous	0.88 (0.71–1.07)	0.79 (0.62–0.99)
NSCLC-NOS	0.91 (0.71–1.18)	0.96 (0.74–1.25)
Small-Cell	1.06 (0.86–1.30)	1.04 (0.83–1.29)
Stage^c^		
Localised	1.05 (0.78–1.42)	0.92 (0.63–1.34)
Regional	0.91 (0.76–1.09)	0.87 (0.72–1.06)
Metastatic	1.03 (0.90–1.17)	1.03 (0.90–1.19)
Histology and Stage^d^		
Adenocarcinoma		
Localised	0.92 (0.53–1.59)	0.89 (0.44–1.80)
Regional	1.04 (0.73–1.49)	1.07 (0.73–1.57)
Metastatic	1.10 (0.89–1.35)	1.07 (0.86–1.34)
Squamous		
Localised	1.22 (0.76–1.95)	0.91 (0.49–1.72)
Regional	0.75 (0.55–1.02)	0.67 (0.47–0.95)
Metastatic	0.91 (0.63–1.31)	0.91 (0.62–1.34)
NSCLC-NOS		
Localised	1.14 (0.46–2.82)	1.26 (0.46–3.46)
Regional	0.82 (0.51–1.32)	0.84 (0.51–1.39)
Metastatic	0.95 (0.69–1.30)	1.00 (0.72–1.39)
Small-cell		
Localised	1.09 (0.40–2.96)	1.22 (0.45–3.36)
Regional	1.06 (0.71–1.58)	1.00 (0.65–1.54)
Metastatic	1.13 (0.88–1.46)	1.12 (0.86–1.47)

Hazard ratios (HRs) and 95% confidence intervals (CIs) from Cox regression for pre-diagnostic metformin use compared to no use of any anti-diabetic medication for all-cause death and lung cancer-specific death.

*NSCLC-NOS* non-small cell lung cancer not otherwise specified.

^a^Adjusted for age, gender, smoking, stage, histology, surgery and radiotherapy.

^b^Adjusted for age, gender, smoking, stage, surgery and radiotherapy.

^c^Adjusted for age, gender, smoking, histology, surgery and radiotherapy.

^d^Adjusted for age, gender, smoking, surgery and radiotherapy.

Post-diagnostic metformin use was associated with better survival in all patients (OS: HR = 0.87; 95%CI 0.77–0.98; LCSS: HR = 0.83; 95%CI 0.73–0.95; Table 3), SCC patients (LCSS: HR = 0.75; 95%CI 0.57–0.98), regional stage patients (LCSS: HR = 0.74; 95%CI 0.59–0.94), and regional stage SCC patients (OS: HR = 0.70; 95%CI 0.49–0.99, LSCC: HR = 0.57; 95%CI 0.38–0.86). Interactions with histology (OS: *P* = 0.625, LCSS: *P* = 0.613) and stage (OS: *P* = 0.942, LCSS: *P* = 0.713) were not significant.

**Table 3:** Post-diagnostic metformin use compared to no use of any anti-diabetic medication for all-cause and lung cancer-specific mortality.

Variables	All-cause death	Lung cancer death
	HR (95%CI)	HR (95%CI)
All patients^a^	0.87 (0.77–0.98)	0.83 (0.73–0.95)
Histology^b^		
Adenocarcinoma	0.83 (0.67–1.03)	0.82 (0.65–1.04)
Squamous	0.87 (0.69–1.09)	0.75 (0.57–0.98)
NSCLC-NOS	0.81 (0.61–1.08)	0.81 (0.59–1.10)
Small-Cell	0.95 (0.73–1.22)	0.95 (0.72–1.24)
Stage^c^		
Localised	0.98 (0.73–1.31)	0.87 (0.60–1.26)
Regional	0.82 (0.67–1.01)	0.74 (0.59–0.94)
Metastatic	0.90 (0.75–1.07)	0.91 (0.76–1.09)
Histology and stage^d^		
Adenocarcinoma		
Localised	0.90 (0.48–1.69)	0.91 (0.40–2.03)
Regional	1.00 (0.68–1.48)	0.98 (0.64–1.52)
Metastatic	0.79 (0.59–1.06)	0.78 (0.57–1.07)
Squamous		
Localised	1.16 (0.73–1.83)	0.84 (0.45–1.58)
Regional	0.70 (0.49–0.99)	0.57 (0.38–0.86)
Metastatic	1.06 (0.66–1.69)	1.04 (0.63–1.71)
NSCLC-NOS		
Localised	0.96 (0.50–1.86)	0.88 (0.40–1.92)
Regional	0.69 (0.39–1.24)	0.67 (0.36–1.26)
Metastatic	0.83 (0.56–1.24)	0.89 (0.60–1.34)
Small-cell		
Localised	0.80 (0.29–2.18)	0.96 (0.35–2.64)
Regional	0.77 (0.46–1.29)	0.72 (0.42–1.26)
Metastatic	1.08 (0.79–1.47)	1.10 (0.80–1.52)

Hazard ratios (HRs) and 95% confidence intervals (CIs) from Cox regression for post-diagnostic metformin use compared to no use of any antidiabetic medication for all-cause death and lung cancer-specific death

*NSCLC-NOS* non-small cell lung cancer not otherwise specified.

^a^Adjusted for age, gender, smoking, stage, histology, surgery and radiotherapy.

^b^Adjusted for age, gender, smoking, stage, surgery and radiotherapy.

^c^Adjusted for age, gender, smoking, histology, surgery and radiotherapy.

^d^Adjusted for age, gender, smoking, surgery and radiotherapy.

Further analysis by cumulative use of metformin (Table 4) indicated a dose response relationship for all patients (*P*_trend_ < 0.001 for both OS and LCSS), adenocarcinoma patients (OS: *P*_trend_ = 0.004; LCSS: *P*_trend_ = 0.003), SCC patients (*P*_trend_ < 0.001 for OS and LCSS), patients diagnosed with regional stage LC (*P*_trend_ < 0.001 for OS and LCSS) in addition to patients diagnosed with metastatic stage (*P*_trend_ = 0.002 for OS and LCSS). For the lowest metformin dose category (1–149 g), initial increased LC-specific mortality was observed for all patients, adenocarcinoma and SCC patients. Interactions with histology (OS: *P* = 0.633, LCSS: *P* = 0.318) and stage (OS: *P* = 0.999, LCSS: *P* = 0.991) were not significant. Results for use of other anti-diabetic medications are reported in Supplementary Tables S3, and S4.

**Table 4:** Post-diagnostic cumulative use of metformin for all-cause and lung cancer-specific mortality.

Variables	Metformin doses	Person-years	All-cause death	Lung cancer death
			HR (95%CI)	HR (95%CI)
All patients^a^	No use	30222.76	Reference	Reference
	1–149 g	266.80	1.11 (0.97–1.28)	1.17 (1.01–1.35)
	150–339 g	430.92	0.84 (0.75–0.95)	0.81 (0.71–0.92)
	340–749 g	472.18	0.84 (0.73–0.95)	0.81 (0.70–0.94)
	>749 g	735.56	0.72 (0.61–0.84)	0.69 (0.56–0.82)
	*P* trend		<0.001	<0.001
Histology^b^				
Adenocarcinoma	No use	13099.85	Reference	Reference
	1–149 g	99.23	1.22 (0.97–1.54)	1.36 (1.07–1.74)
	150–339 g	158.70	0.76 (0.61–0.95)	0.74 (0.58–0.95)
	340–749 g	186.03	0.90 (0.72–1.13)	0.83 (0.64–1.06)
	>749 g	296.49	0.70 (0.52–0.93)	0.68 (0.49–0.94)
	*P* trend		0.004	0.003
Squamous	No use	7184.82	Reference	Reference
	1–149 g	66.68	1.36 (0.98–1.87)	1.42 (1.01–2.01)
	150–339 g	126.19	0.87 (0.68–1.13)	0.79 (0.60–1.06)
	340–749 g	145.49	0.73 (0.56–0.95)	0.70 (0.53–0.94)
	>749 g	259.51	0.66 (0.50–0.88)	0.63 (0.45–0.88)
	*P* trend		<0.001	<0.001
NSCLC-NOS	No use	3157.91	Reference	Reference
	1–149 g	29.86	0.88 (0.63–1.23)	0.86 (0.60–1.23)
	150–339 g	40.59	0.91 (0.68–1.22)	0.91 (0.66–1.24)
	340–749 g	32.08	0.96 (0.66–1.38)	0.97 (0.66–1.43)
	>749 g	47.88	0.86 (0.53–1.38)	0.79 (0.47–1.36)
	*P* trend		0.374	0.326
Small-cell	No use	3567.99	Reference	Reference
	1–149 g	50.02	1.11 (0.84–1.46)	1.18 (0.89–1.57)
	150–339 g	77.35	0.87 (0.68–1.11)	0.84 (0.65–1.09)
	340–749 g	72.94	0.96 (0.73–1.26)	0.97 (0.74–1.29)
	>749 g	51.62	1.00 (0.68–1.49)	0.91 (0.59–1.41)
	*P* trend		0.613	0.454
Stage^c^				
Localised	No use	11137.57	Reference	Reference
	1–149 g	97.70	1.14 (0.74–1.76)	1.24 (0.76–2.05)
	150–339 g	131.08	1.00 (0.70–1.42)	0.70 (0.43–1.15)
	340–749 g	177.23	0.83 (0.57–1.19)	0.73 (0.46–1.16)
	>749 g	368.81	0.73 (0.52–1.03)	0.75 (0.49–1.16)
	*P* trend		0.067	0.053
Regional	No use	10621.81	Reference	Reference
	1–149 g	83.67	1.17 (0.90–1.53)	1.16 (0.87–1.54)
	150–339 g	167.81	0.89 (0.71–1.11)	0.88 (0.70–1.12)
	340–749 g	180.51	0.80 (0.63–1.00)	0.76 (0.59–0.97)
	>749 g	232.33	0.65 (0.50–0.84)	0.61 (0.46–0.82)
	*P* trend		<0.001	<0.001
Metastatic	No use	6938.67	Reference	Reference
	1–149 g	68.40	1.12 (0.93–1.33)	1.19 (0.99–1.43)
	150–339 g	117.18	0.81 (0.69–0.95)	0.80 (0.67–0.94)
	340–749 g	88.62	0.84 (0.70–1.02)	0.84 (0.69–1.02)
	>749 g	77.62	0.73 (0.55–0.98)	0.71 (0.52–0.97)
	*P* trend		0.002	0.002

Hazard ratios (HRs) and 95% confidence intervals (CI)s from Cox regression for post-diagnostic cumulative metformin use for all-cause death and lung cancer- specific death.

*NSCLC-NOS* non-small cell lung cancer not otherwise specified.

*P* trend was estimated by analysing the categorical cumulative DDDs as a continuous variable.

^a^Adjusted for age, gender, smoking, stage, histology, surgery, radiotherapy, and use of any other anti-diabetic medication.

^b^Adjusted for age, gender, smoking, stage, surgery, radiotherapy and use of any other anti-diabetic medication.

^c^Adjusted for age, gender, smoking, histology, surgery, radiotherapy and use of any other anti-diabetic medication.

## Discussion

In our large population-based cohort of Norwegian patients, pre-diagnostic metformin use was associated with improved LCSS in patients with SCC histology, especially in those with regional stage. Post-diagnostic metformin use was associated with improved OS and LCSS for LC patients overall, and also with improved LCSS for SCC histology and regional stage. The association between metformin and LCSS was corroborated by a dose-response relationship for cumulative use. This association was not only present for SCC and regional disease, but also for adenocarcinoma and metastatic stage.

Metformin potentially exerts its anti-cancer activity by altering neoplastic cellular energy metabolism in an AMPK (adenosine monophosphate-active protein kinase) -dependent or an AMPK-independent signaling pathway,^3^ inhibiting protein synthesis, stopping the cell cycle, decreasing blood insulin levels, improving glycaemic control and immune system, destroying cancer stem cells and reducing the inflammatory response.^4,5^ These laboratory evidence yielded rationale for clinical studies to advance on investigating the role of metformin in survival for many site-specific cancers, including LC. Clinical series and pharmaco-epidemiological studies have suggested that metformin use is associated with prolonged survival for LC patients, and have later been summarised in systematic reviews and meta-analyses.^27–33^ Many of the individual studies were criticised for small sample size, variation in patient characteristics and LC stages, lack of information about the timing and dose of metformin exposure, inadequate adjustment for confounders, poor choice of outcome measure and the reference group.

We recognise that studying the association between metformin use and LC survival is challenging with an observational study due to the heterogeneity between the metformin group and any possible comparison group. Metformin is a first-line treatment for early-stage T2D, and increasing severity of DM over time often requires a switch to other hypoglycaemic agents or insulin.^32^ To avoid an overestimation of the association between metformin and LC survival, comparing metformin users with non-users of any anti-diabetic medication has been advocated.^6,7,13,20–24,34^ This is also supported by our findings indicating that especially insulin users had a worse survival than the non-anti-diabetic users (Table S4). On the other hand, since DM does not have a major impact on OS in LC patients,^7,34^ comparing diabetic metformin users with non-diabetic non-metformin users should not lead to an important underestimation of the association between metformin use and OS. Based on this, we chose to use non-use of any anti-diabetic medication as the reference group.

The prognostic significance of DM in LC patients is unclear, and conflicting results are reported in the literature.^35^ In our study, it is impossible to disentangle a possible prognostic effect of DM from the one of metformin, since we do not have information about DM diagnosis and because metformin is almost exclusively prescribed for the treatment of T2D. Thus, we cannot rule out that the metformin association is to some extent confounded by DM.

The identification of the impact of metformin in non-diabetic patients can best be ascertained in prospective randomised clinical trials, and investigations of metformin in trials should be alluring because it is a well tolerable and inexpensive drug. Nonetheless, many trials were terminated due to poor accrual and only a few produced results. For patients with advanced NSCLC stage, promising results were reported on the combination of metformin with chemotherapy, albeit with underpowered samples of 14, 25 and 30 patients.^22–24^ For patients with advanced disease and epidermal growth factor receptor (EGFR) mutations, two trials reported conflicting results. Arrieta et al.^20^ found significantly prolonged OS and progression-free survival (PFS) in the metformin plus EGFR-tyrosine kinase inhibitor (TKI) arm compared to the EGFR-TKI alone group with a similar frequency of adverse effects in both groups. Contrary, Li et al.^21^ showed that metformin in addition to EGFR-TKI therapy did not prolong OS and PFS, but increased toxicity.

Observational studies have indicated evidence of improved LC survival among metformin users.^1,6–8,11,13–15,17,18^ Nevertheless, many metformin studies have been criticised for time-related biases, especially immortal time bias. Even if the benefit of use of metformin is replicated in different settings, the association between metformin and LC survival may not be trustworthy when studies are methodologically inaccurate.^26^ Using the time-dependent analysis in our study, the metformin exposure was correctly classified during follow-up. This strategy prevents immortal time bias. Chuang et al.^1^ and Jie Lin et al.^11^ and also applied Cox proportional hazard model with a time-dependent covariate, and found survival benefit with metformin use among LC patients.

We acknowledge that our study is the first to report an association between metformin use and prolonged survival for SCC regional stage LC patients. This finding can be supported by existing information about biological mechanisms. Exploiting the LKB1-AMPK-mTOR signaling pathway and by decreasing the ΔNp63α expression, metformin may reduce the proliferation of cancer cells. LKB1 (liver kinase B1) encodes a tumour suppressor that activates AMPK, a molecule that is able to inhibit the mammalian target of rapamycin complex (mTORC 1), leading to reduced protein synthesis in cancer cells.^3,4^ LKB1 is somatically deactivated in 30–50% of lung cancer but in only 20% of SCCs.^4^ Moreover, ΔNp63α is the predominant p63 protein isoform, and has been reported to promote cancer cell proliferation.^36^ ΔNp63α is frequently overexpressed in SCC cells.^36^ By an independent AMPK pathway,^36,37^ metformin may decrease ΔNp63α expression under glucose deprivation.^36^ Regional stage comprises TNM stages II and III. Surgery with adjuvant chemotherapy is the recommended treatment for N0–1, technically operable disease. Otherwise curative radiotherapy, and for stage III in combination with chemotherapy, is standard treatment.^38^ Interaction between metformin use and LC treatment is plausible but the study of effect modification requires larger series or prospective intervention studies. Pharmaco-epidemiological studies are known to be prone to bias, but it is unlikely that bias would be restricted to certain histological types or stage subgroups.

This study has considerable strengths. Due to the nationwide design, we were able to study the use of metformin and LC survival without selection bias and recall bias of drug use. CRN provides cancer data with high quality and completeness which ensures the reliability of our findings. Additionally, LCSS was available in our study. This should be the preferred outcome measure for observational studies, since it reduces the impact of competing causes of death, and has only been reported in one prior study.^39^ Further, we were able to adjust for relevant variables for LC, including smoking. According to Zhang et al.,^40^ the lack of smoking adjustment led to an overestimation of the potential benefit of metformin and it was one of the major sources of heterogeneity between the studies in their meta-analysis. An additional strength is the use of time-dependent analyses. Observational studies have shown improved LC survival with metformin use. However, many metformin studies have been criticised for time-related biases, especially immortal time bias. This bias exaggerates the effect of a drug, thus making a drug seem to be protective when in fact it may have no effect. When estimating the association with cumulative dose of metformin, this becomes especially relevant as patients who live longer have the possibility to take higher cumulative doses. Using the time-dependent analysis in our study, the metformin exposure was correctly classified during follow-up.

We also highlight our limitations. Firstly, the diagnosis of DM was not clinically defined. Thus, we presumed that metformin users were diabetic patients as metformin is almost exclusively used for the treatment of T2D. Secondly, we do not know how patient compliance was and if the patient actually took metformin after receiving the prescription. On the other hand, since a drug prescription was the medical option it is unlikely that a diabetic patient did not use the repeatedly dispensed drug for controlling DM. Thirdly, the duration of metformin use was not available. The sum of consumption of metformin during the study period was used as a measure instead. Fourth, there is a possibility that metformin was underestimated in the older patients as drugs given in institutions and retirement homes are not registered in the prescription database. This would result in a miss-classification of the oldest patients which would weaken the association. Fifth, comorbidities and chemotherapy were not appraised by our study. Regarding comorbidities, the topic is controversial since comorbid conditions might contribute to worse LC survival^41^ but, on the other hand, might be the reason for an earlier LC diagnosis due to greater opportunities for screening and higher frequency of physician visits.^42^ Nonetheless, LCSS is considered to be mainly unaffected by the comorbidity burden.^43^ Chemotherapy, as neoadjuvant and adjuvant treatments, is mainly based on the stage of LC.^38^ Since adjusted analyses controlled for LC stage were made, the lack of adjustment for chemotherapy should not be a major problem in our analysis. Sixth, we are aware that death certification is not always a fully reliable source of information in  LC patients. However, the recording accuracy in the cause of death registry in Norway is considered trustworthy.^44^ Seventh, despite the non-significant interactions, we decided to report the subgroup analyses according to stage and histology as an exploration of the effect of metformin in different subgroups of tumours. Finally, adjustment for multiple testing was not applied as we consider the stratified analyses to be predefined and explorative.^45^

## Conclusions

In this large Norwegian population-based cohort study, metformin use was associated with improved survival for LC patients, especially LCSS within SCC regional stage. However, prospective studies are needed to fully elucidate the potential benefits of metformin use as an add-on drug for LC.

## Disclaimer

Where authors are identified as personnel of the International Agency for Research on Cancer/World Health Organization, the authors alone are responsible for the views expressed in this article and they do not necessarily represent the decisions, policy or views of the International Agency for Research on Cancer / World Health Organization.

## Supplementary information

Supplementary Tables

## Data Availability

The data that support the findings of this study are available from The Norwegian Institute of Public Health but restrictions apply to the availability of these data, which were used under license for the current study, and so are not publicly available. Data are, however, available from the authors upon reasonable request and with permission of The Norwegian Institute of Public Health.
