# Supplementary material for: Metformin use and lung cancer survival: a population-based study in Norway
Source: Br J Cancer. 2020 Dec 2;124(5):1018–25. doi: 10.1038/s41416-020-01186-9 (PMC7921644; doi:10.1038/s41416-020-01186-9)
Supplement: Supplementary file 1 — Supplementary Tables [file 41416_2020_1186_MOESM1_ESM.docx]

SUPLEMENTARY TABLES

Table S1. Characteristics of lung cancer patients at baseline

| Characteristics | No use ^a^ | Metformin | *P* value | Insulin | Other | Combination | *P* value between all groups |
| --- | --- | --- | --- | --- | --- | --- | --- |
| Total (%) | 20565 (92.1) | 560 (2.5) |  | 303 (1.4) | 198 (0.9) | 698 (3.1) |  |
| Age (y) |  |  | <0.001 ^b^ |  |  |  | <0.001 ^b^ |
| *<60* | 3944 (19.2) | 59 (10.5) |  | 44 (14.5) | 13 (6.6) | 65 (9.3) |  |
| *60-69* | 7051 (34.3) | 208 (37.1) |  | 122 (40.3) | 47 (23.7) | 248 (35.5) |  |
| *70-79* | 6718 (32.7) | 212 (38.0) |  | 101 (33.3) | 88 (44.4) | 297 (42.6) |  |
| *80 and older* | 2852 (13.9) | 81 (14.4) |  | 36 (11.9) | 50 (25.3) | 88 (12.6) |  |
| Gender |  |  | 0.004 |  |  |  | <0.001 |
| *Male* | 11378 (55.3) | 345 (61.6) |  | 201 (66.3) | 136 (68.7) | 463 (66.3) |  |
| *Female* | 9187 (44.7) | 215 (38.4) |  | 102 (33.7) | 62 (31.3) | 235 (33.7) |  |
| Smoking |  |  | 0.006 |  |  |  | <0.001 |
| *Current* | 8186 (56.9) | 196 (49.1) |  | 109 (52.2) | 68 (50.0) | 226 (47.8) |  |
| *Previous* | 4839 (33.6) | 163 (40.9) |  | 82 (39.3) | 54 (39.7) | 208 (44.0) |  |
| *Never* | 1360 (9.5) | 40 (10.0) |  | 18 (8.6) | 14 (10.3) | 39 (8.3) |  |
| *Missing* | 6180 | 161 |  | 94 | 62 | 225 |  |
| Stage |  |  | 0.711 |  |  |  | 0.004 |
| *Localized* | 4218 (21.3) | 113 (20.7) |  | 76 (26.4) | 55 (29.3) | 139 (20.7) |  |
| *Regional* | 6173 (31.1) | 179 (32.8) |  | 74 (25.7) | 70 (37.2) | 216 (32.1) |  |
| *Metastatic* | 9441 (47.6) | 254 (46.5) |  | 138 (47.9) | 63 (33.5) | 317 (47.2) |  |
| *Missing* | 733 | 14 |  | 15 | 10 | 26 |  |
| Histology |  |  | 0.019 ^c^ |  |  |  | <0.001 ^c^ |
| *Adenocarcinoma* | 8287 (40.3) | 198 (35.4) |  | 108 (35.6) | 72 (36.4) | 242 (34.7) |  |
| *Squamous* | 4507 (21.9) | 141 (25.2) |  | 83 (27.4) | 55 (27.8) | 174 (24.9) |  |
| *NSCLC-NOS* | 2971 (14.5) | 69 (12.3) |  | 29 (9.6) | 32 (16.2) | 80 (11.5) |  |
| *Large-Cell* | 391 (1.9) | 8 (1.4) |  | 2 (0.7) | 2 (1.0) | 8 (1.2) |  |
| *Small-Cell* | 3431 (16.7) | 114 (20.4) |  | 59 (19.5) | 27 (13.6) | 153 (21.9) |  |
| *Others* | 978 (4.8) | 30 (5.4) |  | 22 (7.3) | 10 (5.1) | 41 (5.9) |  |
| Surgery |  |  | 0.449 |  |  |  | 0.122 |
| *No Curative-Intent* | 15480 (75.3) | 430 (76.8) |  | 240 (79.2) | 157 (79.3) | 545 (78.1) |  |
| *Curative-Intent* | 5079 (24.7) | 130 (23.2) |  | 63 (20.8) | 41 (20.7) | 153 (21.9) |  |
| *Missing* | 6 | 0 |  | 0 | 0 | 0 |  |
| Radiotherapy |  |  | 0.214 |  |  |  | <0.001 |
| *No* | 10121 (49.2) | 291 (52.2) |  | 164 (54.1) | 110 (55.6) | 392 (56.2) |  |
| *Only Curative-Intent* | 1899 (9.2) | 56 (10.0) |  | 28 (9.2) | 25 (12.6) | 74 (10.6) |  |
| *Other Intent* | 8545 (41.6) | 212 (37.9) |  | 111 (36.6) | 63 (31.9) | 232 (33.2) |  |

^a^ No use of any anti-diabetic medication

^b^ *P* value based on categorical age

^c^ From Fisher´s exact test

*P* values do not include missing category

NSCLC-NOS: Non-Small Cell Lung Cancer Not Otherwise Specified

Table S2. Pre-diagnostic antidiabetic medication use for all-cause and lung cancer-specific mortality

| **VARIABLES** | **ALL-CAUSE DEATH** | |  | **LUNG CANCER DEATH** | |
| --- | --- | --- | --- | --- | --- |
|  | **ANTIDIABETIC MEDICATION** | **HR (95% CI)** |  | **ANTIDIABETIC MEDICATION** | **HR (95% CI)** |
|  |  |  |  |  |  |
| ***ALL PATIENTS*** ^a^ | NO MEDICATION | REFERENCE |  | NO MEDICATION | REFERENCE |
|  | METFORMIN | 0.97 (0.88- 1.07) |  | METFORMIN | 0.95 (0.86-1.06) |
|  | INSULIN | 1.08 (0.95-1.22) |  | INSULIN | 1.03 (0.89-1.18) |
|  | OTHER | 1.12 (0.96-1.31) |  | OTHER | 1.13 (0.96-1.34) |
|  | COMBINATION | 1.08 (0.99-1.18) |  | COMBINATION | 1.05 (0.95-1.15) |
|  |  |  |  |  |  |
| ***HISTOLOG Y*** ^b^ |  |  |  |  |  |
|  |  |  |  |  |  |
| *ADENOCARCINOMA* | NO MEDICATION | REFERENCE |  | NO MEDICATION | REFERENCE |
|  | METFORMIN | 1.05 (0.89-1.25) |  | METFORMIN | 1.05 (0.87-1.26) |
|  | INSULIN | 1.11 (0.89-1.37) |  | INSULIN | 1.04 (0.82-1.32) |
|  | OTHER | 1.07 (0.82-1.40) |  | OTHER | 1.13 (0.85-1.51) |
|  | COMBINATION | 1.06 (0.91-1.23) |  | COMBINATION | 1.00 (0.85-1.18) |
|  |  |  |  |  |  |
| *SQUAMOUS CELL* | NO MEDICATION | REFERENCE |  | NO MEDICATION | REFERENCE |
|  | METFORMIN | 0.88 (0.71-1.07) |  | METFORMIN | 0.79 (0.62-0.99) |
|  | INSULIN | 0.99 (0.78-1.26) |  | INSULIN | 0.93 (0.71-1.23) |
|  | OTHER | 1.19 (0.89-1.59) |  | OTHER | 1.16 (0.84-1.59) |
|  | COMBINATION | 1.03 (0.86-1.23) |  | COMBINATION | 1.01 (0.83-1.23) |
|  |  |  |  |  |  |
| *NSCLC-NOS* | NO MEDICATION | REFERENCE |  | NO MEDICATION | REFERENCE |
|  | METFORMIN | 0.91 (0.71-1.18) |  | METFORMIN | 0.96 (0.74-1.25) |
|  | INSULIN | 0.98 (0.66-1.44) |  | INSULIN | 1.01 (0.68-1.52) |
|  | OTHER | 1.00 (0.69-1.45) |  | OTHER | 0.96 (0.64-1.45) |
|  | COMBINATION | 1.05 (0.84-1.33) |  | COMBINATION | 1.01 (0.78-1.30) |
|  |  |  |  |  |  |
| *SMALL CELL* | NO MEDICATION | REFERENCE |  | NO MEDICATION | REFERENCE |
|  | METFORMIN | 1.06 (0.86-1.30) |  | METFORMIN | 1.04 (0.83-1.29) |
|  | INSULIN | 1.06 (0.80-1.40) |  | INSULIN | 1.02 (0.76-1.38) |
|  | OTHER | 0.96 (0.64-1.44) |  | OTHER | 0.97 (0.64-1.49) |
|  | COMBINATION | 1.29 (1.08-1.53) |  | COMBINATION | 1.26 (1.05-1.52) |
|  |  |  |  |  |  |
| ***STAGE*** ^c^ |  |  |  |  |  |
|  |  |  |  |  |  |
| *LOCALIZED* | NO MEDICATION | REFERENCE |  | NO MEDICATION | REFERENCE |
|  | METFORMIN | 1.05 (0.78-1.42) |  | METFORMIN | 0.92 (0.63-1.34) |
|  | INSULIN | 1.27 (0.92-1.75) |  | INSULIN | 1.30 (0.90-1.88) |
|  | OTHER | 1.14 (0.82-1.60) |  | OTHER | 1.20 (0.81-1.77) |
|  | COMBINATION | 1.00 (0.77-1.30) |  | COMBINATION | 0.86 (0.62-1.20) |
|  |  |  |  |  |  |
| *REGIONAL* | NO MEDICATION | REFERENCE |  | NO MEDICATION | REFERENCE |
|  | METFORMIN | 0.91 (0.76-1.09) |  | METFORMIN | 0.87 (0.72-1.06) |
|  | INSULIN | 0.81 (0.62-1.06) |  | INSULIN | 0.71 (0.52-0.96) |
|  | OTHER | 1.23 (0.95-1.60) |  | OTHER | 1.20 (0.90-1.59) |
|  | COMBINATION | 1.02 (0.87-1.19) |  | COMBINATION | 0.99 (0.83-1.17) |
|  |  |  |  |  |  |
| *METASTATIC* | NO MEDICATION | REFERENCE |  | NO MEDICATION | REFERENCE |
|  | METFORMIN | 1.03 (0.90-1.17) |  | METFORMIN | 1.03 (0.90-1.19) |
|  | INSULIN | 1.14 (0.96-1.36) |  | INSULIN | 1.13 (0.94-1.36) |
|  | OTHER | 0.95 (0.74-1.23) |  | OTHER | 0.97 (0.74-1.27) |
|  | COMBINATION | 1.09 (0.97-1.23) |  | COMBINATION | 1.07 (0.94-1.21) |
|  |  |  |  |  |  |
| ***HISTOLOGY AND STAGE*** ^d^ |  |  |  |  |  |
|  |  |  |  |  |  |
| ***ADENOCARCINOMA*** |  |  |  |  |  |
| **LOCALIZED** | NO MEDICATION | REFERENCE |  | NO MEDICATION | REFERENCE |
|  | METFORMIN | 0.92 (0.53-1.59) |  | METFORMIN | 0.89 (0.44-1.80) |
|  | INSULIN | 1.93 (1.19-3.14) |  | INSULIN | 2.25 ( 1.29-3.94) |
|  | OTHER | 0.90 (0.45-1.81) |  | OTHER | 0.73 (0.27-1.96) |
|  | COMBINATION | 1.13 (0.70-1.84) |  | COMBINATION | 1.08 (0.59-1.97) |
|  |  |  |  |  |  |
| **REGIONAL** | NO MEDICATION | REFERENCE |  | NO MEDICATION | REFERENCE |
|  | METFORMIN | 1.04 (0.73-1.49) |  | METFORMIN | 1.07 (0.73-1.57) |
|  | INSULIN | 0.81 (0.50-1.31) |  | INSULIN | 0.54 (0.29-1.01) |
|  | OTHER | 1.34 (0.86-2.11) |  | OTHER | 1.47 (0.91-2.36) |
|  | COMBINATION | 1.04 (0.78-1.39) |  | COMBINATION | 0.99 (0.72-1.35) |
|  |  |  |  |  |  |
| **METASTATIC** | NO MEDICATION | REFERENCE |  | NO MEDICATION | REFERENCE |
|  | METFORMIN | 1.10 (0.89-1.35) |  | METFORMIN | 1.07 (0.86-1.34) |
|  | INSULIN | 1.13 (0.84-1.51) |  | INSULIN | 1.13 (0.84-1.54) |
|  | OTHER | 0.88 (0.58-1.32) |  | OTHER | 0.95 (0.62-1.44) |
|  | COMBINATION | 1.07 (0.88-1.31) |  | COMBINATION | 1.02 (0.82-1.26) |
|  |  |  |  |  |  |
| ***SQUAMOUS-CELL*** |  |  |  |  |  |
| **LOCALIZED** | NO MEDICATION | REFERENCE |  | NO MEDICATION | REFERENCE |
|  | METFORMIN | 1.22 (0.76-1.95) |  | METFORMIN | 0.91 (0.49-1.72) |
|  | INSULIN | 0.97 (0.58-1.63) |  | INSULIN | 0.92 ( 0.50-1.68) |
|  | OTHER | 1.67 (1.03-2.69) |  | OTHER | 2.07 (1.22-3.50) |
|  | COMBINATION | 0.83 (0.54-1.29) |  | COMBINATION | 0.50 (0.26-0.96) |
|  |  |  |  |  |  |
| **REGIONAL** | NO MEDICATION | REFERENCE |  | NO MEDICATION | REFERENCE |
|  | METFORMIN | 0.75 (0.55-1.02) |  | METFORMIN | 0.67 (0.47-0.95) |
|  | INSULIN | 0.92 (0.63-1.36) |  | INSULIN | 0.90 (0.59-1.36) |
|  | OTHER | 1.21 (0.77-1.92) |  | OTHER | 0.97 (0.57-1.66) |
|  | COMBINATION | 0.84 (0.64-1.11) |  | COMBINATION | 0.84 (0.63-1.13) |
|  |  |  |  |  |  |
| **METASTATIC** | NO MEDICATION | REFERENCE |  | NO MEDICATION | REFERENCE |
|  | METFORMIN | 0.91 (0.63-1.31) |  | METFORMIN | 0.91 (0.62-1.34) |
|  | INSULIN | 0.99 (0.64-1.53) |  | INSULIN | 0.90 (0.55-1.45) |
|  | OTHER | 0.87 (0.43-1.74) |  | OTHER | 0.95 (0.47-1.91) |
|  | COMBINATION | 1.42 (1.05-1.91) |  | COMBINATION | 1.51 (1.11-2.06) |
|  |  |  |  |  |  |
| ***NSCLC-NOS*** |  |  |  |  |  |
| **LOCALIZED** | NO MEDICATION | REFERENCE |  | NO MEDICATION | REFERENCE |
|  | METFORMIN | 1.14 (0.46-2.82) |  | METFORMIN | 1.26 (0.46-3.46) |
|  | INSULIN | 0.57 (0.08-4.12) |  | INSULIN | 0.80 (0.11- 5.84) |
|  | OTHER | 0.95 (0.42-2.14) |  | OTHER | 0.81 (0.30-2.21) |
|  | COMBINATION | 2.25 (0.99-5.15) |  | COMBINATION | 1.99 (0.72-5.45) |
|  |  |  |  |  |  |
| **REGIONAL** | NO MEDICATION | REFERENCE |  | NO MEDICATION | REFERENCE |
|  | METFORMIN | 0.82 (0.51-1.32) |  | METFORMIN | 0.84 (0.51-1.39) |
|  | INSULIN | 0.83 (0.31-2.23) |  | INSULIN | 0.99 (0.37-2.68) |
|  | OTHER | 1.03 (0.53-2.00) |  | OTHER | 1.17 (0.60-2.27) |
|  | COMBINATION | 0.77 (0.51-1.17) |  | COMBINATION | 0.72 ( 0.46-1.12) |
|  |  |  |  |  |  |
| **METASTATIC** | NO MEDICATION | REFERENCE |  | NO MEDICATION | REFERENCE |
|  | METFORMIN | 0.95 (0.69-1.30) |  | METFORMIN | 1.00 (0.72-1.39) |
|  | INSULIN | 1.03 (0.65-1.64) |  | INSULIN | 1.11 (0.70-1.78) |
|  | OTHER | 1.03 (0.59-1.80) |  | OTHER | 0.89 ( 0.48-1.68) |
|  | COMBINATION | 1.09 (0.80-1.51) |  | COMBINATION | 1.06 (0.75-1.49) |
|  |  |  |  |  |  |
| ***SMALL-CELL*** |  |  |  |  |  |
| **LOCALIZED** | NO MEDICATION | REFERENCE |  | NO MEDICATION | REFERENCE |
|  | METFORMIN | 1.09 (0.40-2.96) |  | METFORMIN | 1.22 (0.45-3.36) |
|  | INSULIN | 2.51 (0.78-8.13) |  | INSULIN | 1.91 (0.46-7.96) |
|  | OTHER | 0.48 (0.12-1.97) |  | OTHER | 0.53 (0.13-2.18) |
|  | COMBINATION | 1.24 (0.69-2.25) |  | COMBINATION | 1.24 (0.65-2.38) |
|  |  |  |  |  |  |
| **REGIONAL** | NO MEDICATION | REFERENCE |  | NO MEDICATION | REFERENCE |
|  | METFORMIN | 1.06 (0.71-1.58) |  | METFORMIN | 1.00 (0.65-1.54) |
|  | INSULIN | 0.63 (0.28-1.43) |  | INSULIN | 0.48 (0.18-1.29) |
|  | OTHER | 1.92 (0.89-4.15) |  | OTHER | 1.82 (0.79-4.18) |
|  | COMBINATION | 1.78 (1.24-2.57) |  | COMBINATION | 1.85 (1.27-2.71) |
|  |  |  |  |  |  |
| **METASTATIC** | NO MEDICATION | REFERENCE |  | NO MEDICATION | REFERENCE |
|  | METFORMIN | 1.13 (0.88-1.46) |  | METFORMIN | 1.12 (0.86-1.47) |
|  | INSULIN | 1.08 (0.78-1.49) |  | INSULIN | 1.08 (0.77-1.51) |
|  | OTHER | 0.88 (0.48-1.59) |  | OTHER | 0.87 (0.46-1.63) |
|  | COMBINATION | 1.24 (1.00-1.55) |  | COMBINATION | 1.21 (0.96-1.53) |

Hazard ratios (HR)s and 95% confidence intervals (CI)s from Cox regression for pre-diagnostic antidiabetic medication use compared to no use of any antidiabetic medication for all-cause death and lung cancer- specific death

^a^ Adjusted for age, gender, smoking, stage, histology, surgery and radiotherapy

^b^ Adjusted for age, gender, smoking, stage, surgery and radiotherapy

^c^ Adjusted for age, gender, smoking, histology, surgery and radiotherapy

^d^ Adjusted for age, gender, smoking, surgery and radiotherapy

NSCLC-NOS: Non-Small Cell Lung Cancer Not Otherwise Specified

Table S3. Post-diagnostic antidiabetic medication use for all-cause and lung cancer-specific mortality

| **VARIABLES** | **ALL-CAUSE DEATH** | |  | **LUNG CANCER DEATH** | |
| --- | --- | --- | --- | --- | --- |
|  | **ANTIDIABETIC MEDICATION** | **HR (95% CI)** |  | **ANTIDIABETIC MEDICATION** | **HR (95% CI)** |
|  |  |  |  |  |  |
| ***ALL PATIENTS*** ^a^ | NO MEDICATION | REFERENCE |  | NO MEDICATION | REFERENCE |
|  | METFORMIN | 0.87 (0.77-0.98) |  | METFORMIN | 0.83 (0.73-0.95) |
|  | INSULIN | 1.40 (1.27-1.55) |  | INSULIN | 1.38 (1.24-1.54) |
|  | OTHER | 1.00 (0.85-1.17) |  | OTHER | 0.99 (0.83-1.19) |
|  | COMBINATION | 1.15 (1.06-1.25) |  | COMBINATION | 1.12 (1.02-1.23) |
|  |  |  |  |  |  |
| ***HISTOLOGY*** ^b^ |  |  |  |  |  |
|  |  |  |  |  |  |
| *ADENOCARCINOMA* | NO MEDICATION | REFERENCE |  | NO MEDICATION | REFERENCE |
|  | METFORMIN | 0.83 (0.67-1.03) |  | METFORMIN | 0.82 (0.64-1.04) |
|  | INSULIN | 1.53 (1.30-1.81) |  | INSULIN | 1.46 (1.22-1.75) |
|  | OTHER | 1.03 (0.78-1.36) |  | OTHER | 1.09 (0.81-1.47) |
|  | COMBINATION | 1.16 (1.00-1.34) |  | COMBINATION | 1.14 (0.97-1.34) |
|  |  |  |  |  |  |
| *SQUAMOUS CELL* | NO MEDICATION | REFERENCE |  | NO MEDICATION | REFERENCE |
|  | METFORMIN | 0.87 (0.69-1.09) |  | METFORMIN | 0.75 (0.57-0.98) |
|  | INSULIN | 1.19 (0.97-1.47) |  | INSULIN | 1.15 (0.91-1.44) |
|  | OTHER | 0.92 (0.67-1.25) |  | OTHER | 0.87 (0.62-1.24) |
|  | COMBINATION | 1.08 (0.90-1.29) |  | COMBINATION | 1.02 (0.83-1.25) |
|  |  |  |  |  |  |
| *NSCLC-NOS* | NO MEDICATION | REFERENCE |  | NO MEDICATION | REFERENCE |
|  | METFORMIN | 0.81 (0.61-1.08) |  | METFORMIN | 0.81 (0.59-1.10) |
|  | INSULIN | 1.31 (0.97-1.77) |  | INSULIN | 1.36 (1.00-1.85) |
|  | OTHER | 0.90 (0.60-1.34) |  | OTHER | 0.95 (0.63-1.45) |
|  | COMBINATION | 1.14 (0.90-1.43) |  | COMBINATION | 1.12 (0.87-1.44) |
|  |  |  |  |  |  |
| *SMALL CELL* | NO MEDICATION | REFERENCE |  | NO MEDICATION | REFERENCE |
|  | METFORMIN | 0.95 (0.73-1.22) |  | METFORMIN | 0.95 (0.72-1.24) |
|  | INSULIN | 1.40 (1.12-1.75) |  | INSULIN | 1.43 (1.13-1.81) |
|  | OTHER | 1.05 (0.73-1.52) |  | OTHER | 0.93 (0.62-1.41) |
|  | COMBINATION | 1.28 (1.07-1.52) |  | COMBINATION | 1.27 (1.06-1.52) |
|  |  |  |  |  |  |
| ***STAGE*** ^c^ |  |  |  |  |  |
|  |  |  |  |  |  |
| *LOCALIZED* | NO MEDICATION | REFERENCE |  | NO MEDICATION | REFERENCE |
|  | METFORMIN | 0.98 (0.73-1.31) |  | METFORMIN | 0.87 (0.60-1.26) |
|  | INSULIN | 1.33 (0.99-1.78) |  | INSULIN | 1.23 (0.86-1.77) |
|  | OTHER | 0.96 (0.65-1.40) |  | OTHER | 0.92 (0.58-1.47) |
|  | COMBINATION | 1.10 (0.87-1.38) |  | COMBINATION | 0.96 (0.72-1.29) |
|  |  |  |  |  |  |
| *REGIONAL* | NO MEDICATION | REFERENCE |  | NO MEDICATION | REFERENCE |
|  | METFORMIN | 0.82 (0.67-1.01) |  | METFORMIN | 0.74 (0.59-0.94) |
|  | INSULIN | 1.24 (1.02-1.52) |  | INSULIN | 1.19 (0.96-1.48) |
|  | OTHER | 1.11 (0.81-1.51) |  | OTHER | 1.03 (0.73-1.46) |
|  | COMBINATION | 1.12 (0.96-1.30) |  | COMBINATION | 1.12 (0.95-1.31) |
|  |  |  |  |  |  |
| *METASTATIC* | NO MEDICATION | REFERENCE |  | NO MEDICATION | REFERENCE |
|  | METFORMIN | 0.90 (0.75-1.07) |  | METFORMIN | 0.91 (0.76-1.09) |
|  | INSULIN | 1.46 (1.28-1.67) |  | INSULIN | 1.47 (1.28-1.69) |
|  | OTHER | 0.98 (0.78-1.24) |  | OTHER | 1.02 (0.81-1.30) |
|  | COMBINATION | 1.12 (0.99-1.26) |  | COMBINATION | 1.10 (0.97-1.25) |
|  |  |  |  |  |  |
| ***HISTOLOGY AND STAGE*** ^d^ |  |  |  |  |  |
|  |  |  |  |  |  |
| ***ADENOCARCINOMA*** |  |  |  |  |  |
| **LOCALIZED** | NO MEDICATION | REFERENCE |  | NO MEDICATION | REFERENCE |
|  | METFORMIN | 0.90 (0.48-1.69) |  | METFORMIN | 0.91 (0.39-2.03) |
|  | INSULIN | 2.39 (1.53-3.74) |  | INSULIN | 1.93 ( 1.06-3.52) |
|  | OTHER | 0.96 (0.47-1.93) |  | OTHER | 1.14 (0.51-2.57) |
|  | COMBINATION | 1.50 (1.01-2.22) |  | COMBINATION | 1.61 (1.00-2.59) |
|  |  |  |  |  |  |
| **REGIONAL** | NO MEDICATION | REFERENCE |  | NO MEDICATION | REFERENCE |
|  | METFORMIN | 1.00 (0.68-1.48) |  | METFORMIN | 0.98 (0.64-1.52) |
|  | INSULIN | 1.35 (0.97-1.87) |  | INSULIN | 1.15 (0.79-1.67) |
|  | OTHER | 1.44 (0.74-2.78) |  | OTHER | 1.15 (0.51-2.57) |
|  | COMBINATION | 1.05 (0.81-1.37) |  | COMBINATION | 1.10 (0.83-1.45) |
|  |  |  |  |  |  |
| **METASTATIC** | NO MEDICATION | REFERENCE |  | NO MEDICATION | REFERENCE |
|  | METFORMIN | 0.79 (0.59-1.06) |  | METFORMIN | 0.78 (0.57-1.07) |
|  | INSULIN | 1.51 (1.22-1.88) |  | INSULIN | 1.57 (1.26-1.96) |
|  | OTHER | 0.98 (0.69-1.39) |  | OTHER | 1.09 (0.76-1.55) |
|  | COMBINATION | 1.15 (0.94-1.41) |  | COMBINATION | 1.09 (0.87-1.36) |
|  |  |  |  |  |  |
| ***SQUAMOUS-CELL*** |  |  |  |  |  |
| **LOCALIZED** | NO MEDICATION | REFERENCE |  | NO MEDICATION | REFERENCE |
|  | METFORMIN | 1.16 (0.73-1.83) |  | METFORMIN | 0.84 (0.45-1.58) |
|  | INSULIN | 0.96 (0.58-1.59) |  | INSULIN | 0.97 ( 0.55-1.73) |
|  | OTHER | 1.04 (0.57-1.90) |  | OTHER | 1.04 (0.49-2.20) |
|  | COMBINATION | 0.89 (0.61-1.29) |  | COMBINATION | 0.60 (0.34-1.04) |
|  |  |  |  |  |  |
| **REGIONAL** | NO MEDICATION | REFERENCE |  | NO MEDICATION | REFERENCE |
|  | METFORMIN | 0.70 (0.49-0.99) |  | METFORMIN | 0.57 (0.38-0.86) |
|  | INSULIN | 1.17 (0.83-1.64) |  | INSULIN | 1.15 (0.80-1.66) |
|  | OTHER | 0.98 (0.58-1.64) |  | OTHER | 0.87 (0.49-1.54) |
|  | COMBINATION | 1.02 (0.78-1.33) |  | COMBINATION | 1.03 (0.78-1.36) |
|  |  |  |  |  |  |
| **METASTATIC** | NO MEDICATION | REFERENCE |  | NO MEDICATION | REFERENCE |
|  | METFORMIN | 1.06 (0.66-1.69) |  | METFORMIN | 1.04 (0.63-1.71) |
|  | INSULIN | 1.19 (0.85-1.66) |  | INSULIN | 1.04 (0.71-1.51) |
|  | OTHER | 0.85 (0.47-1.54) |  | OTHER | 0.94 (0.52-1.71) |
|  | COMBINATION | 1.27 (0.91-1.76) |  | COMBINATION | 1.26 (0.89-1.79) |
|  |  |  |  |  |  |
| ***NSCLC-NOS*** |  |  |  |  |  |
| **LOCALIZED** | NO MEDICATION | REFERENCE |  | NO MEDICATION | REFERENCE |
|  | METFORMIN | 0.96 (0.50-1.86) |  | METFORMIN | 0.88 (0.40-1.92) |
|  | INSULIN | 0.45 (0.06-3.26) |  | INSULIN | 0.63 (0.09-4.55) |
|  | OTHER | 0.75 (0.31-1.84) |  | OTHER | 0.78 (0.29-2.23) |
|  | COMBINATION | 2.30 (1.06-4.95) |  | COMBINATION | 1.70 ( 0.62-4.68) |
|  |  |  |  |  |  |
| **REGIONAL** | NO MEDICATION | REFERENCE |  | NO MEDICATION | REFERENCE |
|  | METFORMIN | 0.69 (0.39-1.24) |  | METFORMIN | 0.67 (0.36-1.26) |
|  | INSULIN | 1.72 (0.96-3.06) |  | INSULIN | 1.96 (1.10-3.49) |
|  | OTHER | 1.34 (0.71-2.51) |  | OTHER | 1.50 (0.80-2.82) |
|  | COMBINATION | 0.73 (0.47-1.13) |  | COMBINATION | 0.70 ( 0.44-1.13) |
|  |  |  |  |  |  |
| **METASTATIC** | NO MEDICATION | REFERENCE |  | NO MEDICATION | REFERENCE |
|  | METFORMIN | 0.83 (0.56-1.24) |  | METFORMIN | 0.89 (0.60-1.34) |
|  | INSULIN | 1.32 (0.91-1.92) |  | INSULIN | 1.38 (0.94-2.02) |
|  | OTHER | 0.81 (0.42-1.57) |  | OTHER | 0.80 ( 0.40-1.61) |
|  | COMBINATION | 1.27 (0.93-1.73) |  | COMBINATION | 1.29 (0.93-1.79) |
|  |  |  |  |  |  |
| ***SMALL-CELL*** |  |  |  |  |  |
| **LOCALIZED** | NO MEDICATION | REFERENCE |  | NO MEDICATION | REFERENCE |
|  | METFORMIN | 0.80 (0.29-2.18) |  | METFORMIN | 0.96 (0.35-2.64) |
|  | INSULIN | 1.65 (0.50-5.46) |  | INSULIN | 1.72 (0.41-7.30) |
|  | OTHER | 2.12 (0.51-8.73) |  | OTHER | 1.18 (0.17-8.58) |
|  | COMBINATION | 0.94 (0.51-1.74) |  | COMBINATION | 1.02 (0.53-1.93) |
|  |  |  |  |  |  |
| **REGIONAL** | NO MEDICATION | REFERENCE |  | NO MEDICATION | REFERENCE |
|  | METFORMIN | 0.77 (0.46-1.29) |  | METFORMIN | 0.72 (0.42-1.26) |
|  | INSULIN | 0.98 (0.56-1.71) |  | INSULIN | 0.92 (0.50-1.68) |
|  | OTHER | 1.35 (0.60-3.04) |  | OTHER | 1.04 (0.39-2.80) |
|  | COMBINATION | 1.95 (1.35-2.83) |  | COMBINATION | 1.92 (1.30-2.83) |
|  |  |  |  |  |  |
| **METASTATIC** | NO MEDICATION | REFERENCE |  | NO MEDICATION | REFERENCE |
|  | METFORMIN | 1.08 (0.79-1.47) |  | METFORMIN | 1.10 (0.80-1.52) |
|  | INSULIN | 1.46 (1.12-1.89) |  | INSULIN | 1.52 (1.17-1.98) |
|  | OTHER | 1.05 (0.66-1.67) |  | OTHER | 0.96 (0.57-1.59) |
|  | COMBINATION | 1.24 (0.99-1.54) |  | COMBINATION | 1.24 (0.98-1.56) |

Hazard ratios (HR)s and 95% confidence intervals (CI)s from Cox regression for post-diagnostic antidiabetic medication use compared to no use of any antidiabetic medication for all-cause death and lung cancer-specific death

^a^ Adjusted for age, gender, smoking, stage, histology, surgery and radiotherapy

^b^ Adjusted for age, gender, smoking, stage, surgery and radiotherapy

^c^ Adjusted for age, gender, smoking, histology, surgery and radiotherapy

^d^ Adjusted for age, gender, smoking, surgery and radiotherapy

NSCLC-NOS: Non-Small Cell Lung Cancer Not Otherwise Specified

Table S4. Frequency of pre-diagnostic anti-diabetic medication use.

| **ATC code** | **Anti-diabetic medication** | **Number of patients** |
| --- | --- | --- |
| A10AB01 | insulin for injection, fast acting | 27 |
| A10AB04 | insulin lispro for injection, fast acting | 60 |
| A10AB05 | insulin aspart for injection, fast acting | 193 |
| A10AB06 | insulin glulisine for injection, fast acting | 1 |
| A10AC01 | insulin for injection, intermediate acting | 417 |
| A10AD01 | insulin lispro for injection, intermediate- or long-acting combined with fast acting | 12 |
| A10AD04 | insulin lispro for injection, intermediate- or long-acting combined with fast acting | 12 |
| A10AD05 | insulin aspart for injection, intermediate- or long-acting combined with fast acting | 113 |
| A10AE04 | insulin glargine for injection, long acting | 45 |
| A10AE05 | insulin detemir for injection, long acting | 12 |
| A10BA02 | Metformin | 1188 |
| A10BB01 | Glibenclamide | 29 |
| A10BB07 | Glipizide | 66 |
| A10BB12 | Glimepiride | 537 |
| A10BD03 | metformin and rosiglitazone | 7 |
| A10BD07 | metformin and sitagliptin | 25 |
| A10BD08 | metformin and vildagliptin | 43 |
| A10BF01 | Acarbose | 20 |
| A10BG02 | Rosiglitazone | 20 |
| A10BG03 | Pioglitazone | 11 |
| A10BH01 | Sitagliptin | 40 |
| A10BH02 | Vildagliptin | 8 |
| A10BH03 | Saxagliptin | 10 |
| A10BH05 | Linagliptin | 8 |
| A10BX02 | Repaglinide | 5 |
| A10BX04 | Exenatide | 3 |
| A10BX07 | Liraglutide | 17 |
| A10BX09 | Dapagliflozin | 2 |
| A10BX10 | Lixisenatide | 1 |
